# A Novel Complex of Chitosan–Sodium Carbonate and Its Properties

**DOI:** 10.3390/md16110416

**Published:** 2018-10-30

**Authors:** Jianying Qian, Xiaomeng Wang, Jie Shu, Chang Su, Jinsong Gong, Zhenghong Xu, Jian Jin, Jinsong Shi

**Affiliations:** 1 School of Pharmaceutical Science, Jiangnan University, Wuxi 214122, China; jackieqian@163.com (J.Q.); 17714542165@163.com (X.W.); emilysu1991@126.com (C.S.); gjs713@163.com (J.G.); zhenghxu@jiangnan.edu.cn (Z.X.); 2 College of Chemistry, Chemical Engineering and Materials Science, Testing and Analysis Center, Soochow University, Suzhou 215123, China; shujie@suda.edu.cn

**Keywords:** chitosan, cytotoxicity, polymer film, sodium carbonate, soluble chitosan complex

## Abstract

Chitosan has excellent properties, as it is nontoxic, mucoadhesive, biocompatible, and biodegradable. However, the poor water solubility of chitosan is a major disadvantage. Here, a novel chitosan-sodium carbonate complex was formed by adding a large amount of sodium carbonate to a chitosan/acetic acid solution, which is water-soluble. Fourier transform infrared spectroscopy, energy dispersive spectrometry, scanning electron microscopy, and solid-state nuclear magnetic resonance techniques were used to detect and characterize the aforementioned complex, which appeared to be a neat flake crystal. Solid-state nuclear magnetic resonance (SSNMR) was used to verify the connections between carbonate, sodium ions, and the protonated amino group in chitosan on the basis of ^13^C signals at the chemical shift of 167.745 ppm and 164.743 ppm. Further confirmation was provided by the strong cross-polarization signals identified by the SSNMR 2D ^13^C–^1^H frequency-switched Lee–Goldberg heteronuclear correlation spectrum. The cytotoxicity of a film prepared using this complex was tested using rat fibroblasts. The results show that the film promoted cell proliferation, which provides evidence to support its nontoxicity. The ease of film-forming and the results of cytocompatibility testing suggest that the chitosan-sodium carbonate complex has the potential for use in tissue engineering.

## 1. Introduction

Chitosan (chemical name: polyglucosamine(1-4)-2-amino-β-d-glucose) is obtained by the deacetylation of chitin, and it is widely found in the exoskeleton of crustaceans, e.g., crab shells, lobsters, and shrimp [1]. This natural polymer has excellent properties, as it is nontoxic, mucoadhesive, biocompatible, and biodegradable. Thus, it has received extensive attention from various industries [2,3,4,5,6,7]. 

The poor water solubility of chitosan is a major disadvantage [3,4]. It is slightly soluble in dimethyl sulfoxide and p-toluenesulfonic acid [5], and only dissolves in water under acidic conditions at pH < 6.5. Under acidic conditions, the amino groups on the chitosan chain are protonated and the positive charge is increased; thus, the polysaccharide chains repel each other to achieve a dissolution effect [6,7]. This is the main disadvantage of chitosan when used as a pharmaceutical excipient, especially when the drug is unstable under acidic conditions.

In order to solve this problem, researchers have chemically modified chitosan to improve its solubility. For example, *N*,*N*,*N*-trimethyl chitosan is a quaternized hydrophilic derivative of chitosan [8], sulfonated chitosan is prepared by using 1,3-propane sultone [9], and carboxymethyl chitosan is synthesized under conditions of basification by monochloroacetic acid [10]. Chemical modification can overcome the shortcoming of insolubility. However, the biosafety of the newly synthesized derivatives remains to be verified, as new groups may introduce unknown biological repellency to the compounds. The cytotoxicity, biocompatibility, and biodegradability of the derivatives need experimental verification [11,12].

Before the discovery of alkaline systems, chitosan was dissolved in organic acid solutions, such as acetic acid solutions. However, chitosan is unstable in acid solutions. The glycoside bonds and acetyl groups of chitosan can be hydrolyzed [13], though the rate of hydrolysis in dilute organic acids is fairly slow. In recent years, research has been carried out on the dissolution of chitosan in alkaline systems: in particular, alkali/urea solvents have been proposed. Using a LiOH/NaOH/KOH-urea system and a freeze-melt process, chitosan can be dissolved in an alkaline solvent system to form a uniform solution [14,15]. The intramolecular and intermolecular hydrogen bonds of chitosan are destroyed during the freezing process, and the recovery of hydrogen bonds is further hindered by the alkali and urea during the melting process, thus achieving dissolution [16]. However, this method involves a low temperature (at least −20 °C) and multiple freeze-thaw cycles. 

Combinations of chitin/chitosan and inorganic salts are rarely reported. A novel hybrid material was prepared by the unidirectional crystal growth of CaCO_3_ while the carbamation of chitin served as the template [17]. This chitin derivative was then converted to a gel film by soaking in methanol, immersing in calcium chloride solution, and adding ammonium carbonate vapor to form a chitin-CaCO_3_ complex. A ternary complex between the ion pair Si^2+^CO_3_^2−^ and the amino groups of chitosan was demonstrated [18]. Among these previous reports, the common point is that most of the reactants are carbonates. When the carbonates are salts of two valence metal ions, most of them are insoluble. In light of the LiOH/NaOH/KOH-urea system, it is possible that alkali carbonates can react with chitosan and form soluble complexes. Therefore, we tried to obtain a soluble chitosan complex by adding an alkali carbonate to a chitosan/acetic acid solution.

The main objective of this study was to provide a soluble chitosan-sodium carbonate complex, formed under alkaline conditions, which could be changed back to chitosan using simple treatments to maintain biocompatibility. By adding a large amount of sodium carbonate to a chitosan/acetic acid solution, this complex was obtained and had a pH of 10.1. We then focused our attention to its characteristics, structure, and cytotoxicity in order to gain information for its application in biomedicine.

## 2. Results and Discussion

**Figure 1:**
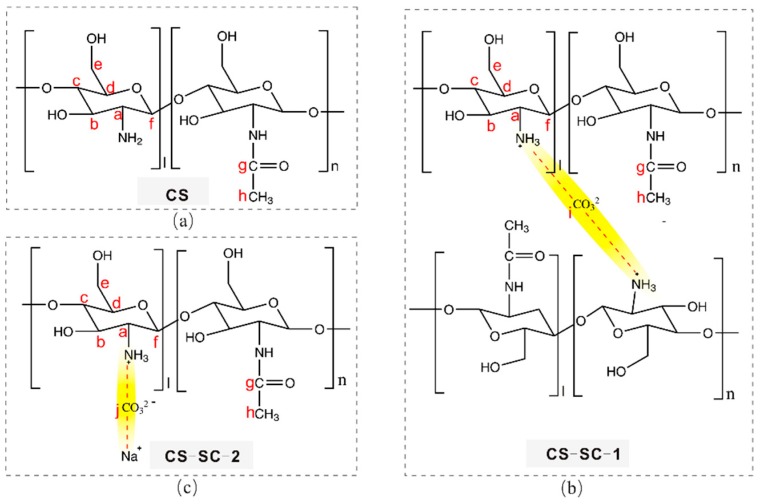
The chemical structural schemes of (**a**) chitosan (CS), (**b**) chitosan-sodium carbonate (CS-SC)-1, and (**c**) CS-SC-2. Carbon atoms are assigned using the alphabetical labels. The connections between carbonate, ammonium, and sodium ions are highlighted in yellow.

The structures of the new compounds forming the chitosan (CS)-sodium carbonate (SC) complex are denoted as CS-SC-1 and CS-SC-2 (Figure 1b,c). A carbonate is bound to either two protonated amino groups to form a complex, or one protonated amino group and one sodium ion. In the figure, the connections between carbonate, ammonium, and sodium ions are highlighted in yellow.

### 2.1. Spectroscopic Investigations

**Figure 2:**
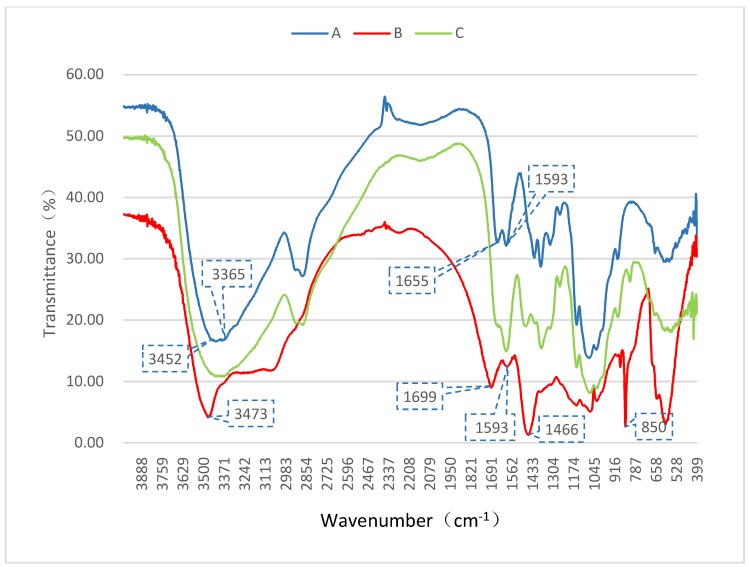
Fourier transform infrared (FT-IR) spectra of chitosan (A, blue), the complex of chitosan-sodium carbonate (B, red), and restored chitosan (C, green).

Fourier transform infrared (FT-IR) spectrometry was used to monitor the structural changes in chitosan (A), the complex of chitosan-sodium carbonate (B), and restored chitosan (C). The obtained infrared spectra are shown in Figure 2. As the amino and hydroxyl groups formed intermolecular and intramolecular hydrogen bonds, the OH stretching vibration and NH stretching vibration, which were originally located around 3400 cm^−1^, fused into a broad intense band at 3452–3365 cm^−1^ in the FT-IR spectrum of chitosan (A), whose characteristic absorption spectrum includes these peaks [19]. The spectrum of the complex (B) shows a completely different absorption peak at 3473 cm^−1^, appearing as a sharp peak, which indicates that the hydrogen bonds between the amino and hydroxyl groups were destroyed, thus showing obvious OH or NH stretching vibrations. The two absorption peaks at 1655 cm^−1^ and 1593 cm^−1^, which are peak I and peak II and represent amide bonds, are also characteristic peaks of chitosan (A) [20]. In the complex (B), these two characteristic peaks still exist but with a slight shift (1699 cm^−1^ and 1593 cm^−1^), indicating the existence of a –NH–CO– structure in the complex. Thus, the residual acetamido in the main chain did not participate in the reaction. The IR spectrum of the complex (B) displayed additional peaks with respect to chitosan (A), which are characteristic peaks of the inserted carbonate group (–CO_3_^2−^) signals: 1466 cm^−1^ and 850 cm^−1^. The strong absorption peaks at these two locations indicate carbonate signals [21].

**Figure 3:**
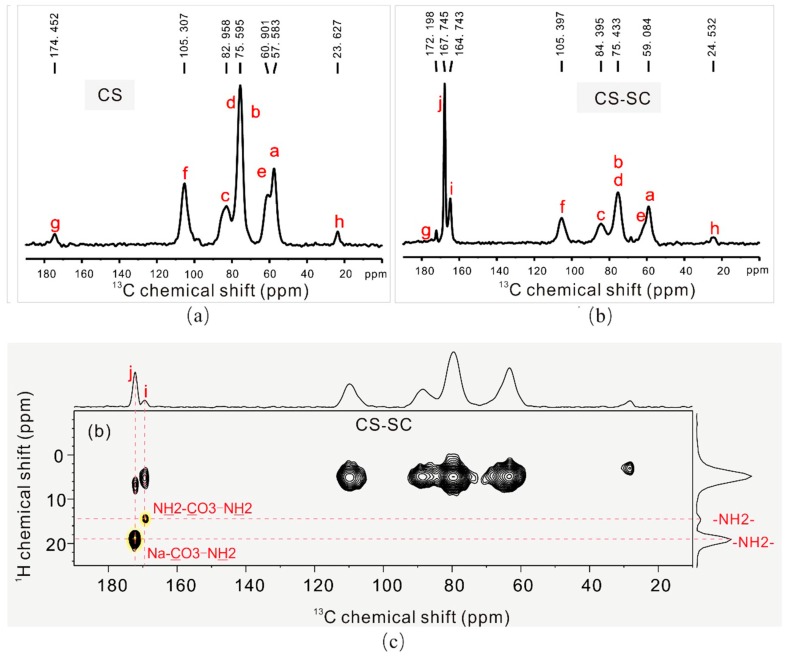
(**a**) Solid-state nuclear magnetic resonance (SSNMR) ^13^C{^1^H} cross-polarization (CP) spectrum of CS. (**b**) SSNMR ^13^C{^1^H} CP spectrum of CS-SC. Peaks are assigned using the alphabetical labels with respect to the structural schemes as shown in Figure 1. (**c**) SSNMR 2D ^13^C–^1^H frequency-switched Lee–Goldberg heteronuclear correlation (FSLG-HETCOR) spectrum of CS-SC, where correlations between CO_3_ and NH_2_ are highlighted in yellow.

Other peaks, such as the CH_3_ symmetrical stretching vibration, CH_2_ asymmetric stretching vibration, CH stretching vibration, CH_2_ bending vibration, CH_3_ deformation vibration, and CO stretching vibration, were similar, proving that the basic structure of chitosan had not been changed, which was also confirmed by the solid-state nuclear magnetic resonance (SSNMR) spectra (in Figure 3). The peaks of C and A are nearly identical, indicating that chitosan could be converted back by adjusting the pH to neutral with hydrochloric acid.

**Figure 4:**
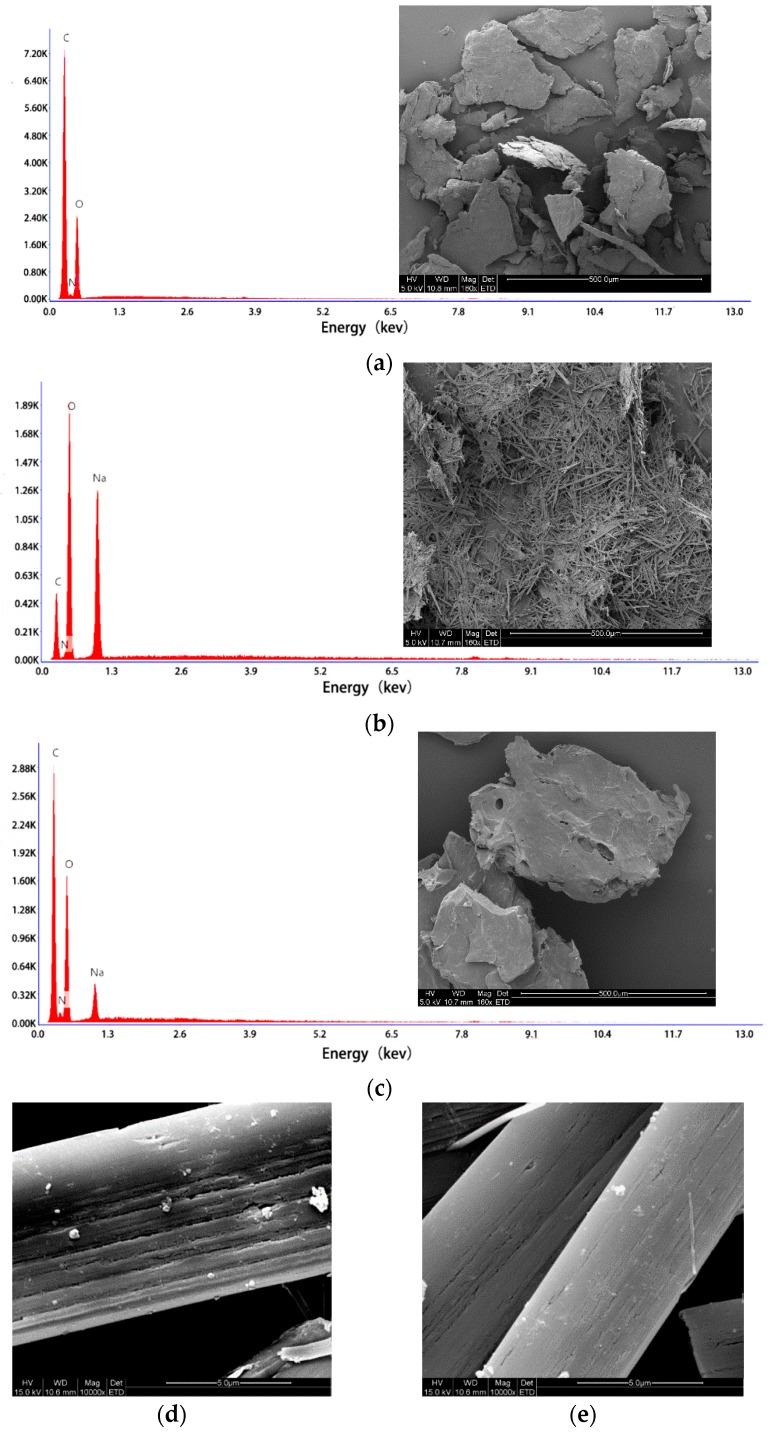
Energy dispersive spectrometry (EDS) spectra and microstructure (magnification 160×, scale bar 500 μm) of chitosan (**a**), the chitosan-sodium carbonate complex (**b**), and restored chitosan (**c**); microstructure (magnification 10,000×, scale bar 5 μm) of the chitosan–sodium carbonate complex (**d**,**e**).

Using a certain beam or monochromatic light (such as X-rays or ultraviolet light) to irradiate the sample, electrons in the atoms or in the molecules on the surface are emitted. The energy distribution of electrons, containing information on and characteristic energy of the surface of the sample, was studied to determine the features of the material surface. The count ratio is affected by the surface roughness of the sample, the angle of photoelectron detection, the X-ray power, and the passing energy; therefore, we could not identify the amount of each element by comparing the size of the peak area [22,23]. Atomic information of chitosan, the chitosan–sodium carbonate complex, and the restored chitosan was analyzed using an energy dispersive spectrometer (EDS) (Figure 4). The atomic percentages (Table 1) reflect the presence or absence of carbonate in the complex. Following the formation of the complex, the atomic percentage of the N atoms decreased significantly and the percentage of Na and O atoms increased, reflecting the addition of carbonate to the mixture. It can be speculated that the carbonate is associated with the amine, which is supported by ionic charges. When the complex was restored to chitosan, the atomic percentage of the N atoms increased because of the dissociation of sodium carbonate and the exposure of NH_2_ to the surface. 

**Figure 5:**
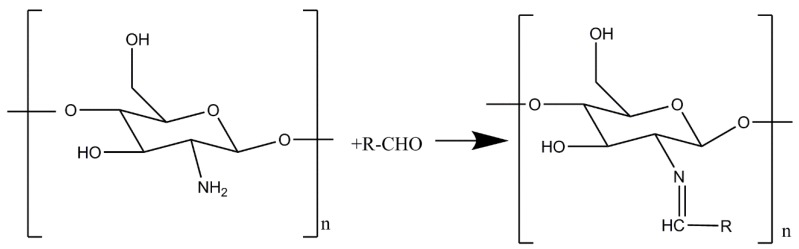
Schiff base reaction.

Chitosan can react with aldehydes (through the Schiff base reaction) by the condensation of amino and aldehyde groups. That is why chitosan can be crosslinked with glutaraldehyde to form a hydrogel [24,25]. The reaction formula is shown in Figure 5. We performed the Schiff base reaction with the chitosan-sodium carbonate complex and glutaraldehyde, adopting a typical preparation process. However, no gel was formed by crosslinking. We prepared restored chitosan by adjusting the solution to pH 5.0 with hydrochloric acid; then, the Schiff base reaction occurred successfully. Therefore, we suppose that the amine of chitosan combined with carbonate during the formation of the complex and dissociated during the restoration.

The microstructure of the three samples was observed by scanning electron microscopy (SEM). Chitosan appeared flake-like when magnified 160×, because the main polysaccharide chain was entangled. The complex formed a rod-like crystal and returned to a mass when adjusted to neutral pH (Figure 4a–c).

Biomacromolecules, such as polysaccharides, proteins, and glycoproteins, can serve as templates, control crystal growth, and thus result in the formation of highly organized complex structures [17]. When the chitosan-sodium carbonate complex was magnified 10,000×, it was observed that the crystalline structure was compact, which was obviously formed by the highly ordered arrangement of molecules (Figure 4d,e). Therefore, we suggest that the chitosan chain acted as a template, the sodium carbonate molecules inserted into it, and a neat crystal structure developed when the complex was formed.

We deduced that the structures of the new compounds that formed the chitosan complex were those of CS-SC-1 and CS-SC-2 in Figure 1b,c. A carbonate is bound to either two protonated amino groups to form a complex, or one protonated amino group and one sodium ion. The connections between carbonate and the sodium ions in the sodium carbonate with the protonated amino group in chitosan were verified by SSNMR (Figure 3), which is able to locally and selectively probe the chemical environment of each carbon nucleus. The identification of each ^13^C peak corresponds with the alphabetical labels shown in Figure 1 and Figure 3. Chitosan (Figure 1a and Figure 3a) was identified by the ^13^C resonances observed at carbon chemical shifts, δ~57.583 (Ca), 75.595 (Cb, Cd), 82.958 (Cc), 60.961 (Ce), 105.307 (Cf) ppm. Two additional ^13^C resonances were observed at δ~174.452 (Cg) and 23.627 (Ch), which were assigned to the residual carbonyl (Cg) and methyl (Ch) typically present in the chitosan skeleton [26,27]. During the reaction of chitosan with sodium carbonate, a chitosan–sodium carbonate complex was obtained (Figure 1b,c), which was composed of CS-SC-1 and CS-SC-2. The structures of CS-SC-1 and CS-SC-2 were easily confirmed by the observation of two ^13^C resonances corresponding to Ci and Cj at δ~164.743 (Ci) and 167.745 (Cj), respectively (Figure 3b). These structures are a consequence of the two possible reactions’ dependence on the amount of sodium carbonate. When the amount of carbonate was insufficient, two ammonium ions bound to a carbonate; when the amount of carbonate was sufficient, one ammonium ion and one sodium ion each bound to carbonate, as shown in Figure 1b,c. It can be seen from the SSNMR 2D ^13^C–^1^H FSLG-HETCOR spectrum of CS-SC (Figure 3c) that the hydrogen on the amino group and the carbon in the carbonyl group had very strong cross-polarization signals, which proves the connection between them.

### 2.2. Cytotoxicity Results

Evaluation of cytotoxicity is an important cell viability method to validate the potential use of the chitosan-sodium carbonate complex in medical bioengineering. As the L929 fibroblast cell line is commonly used to determine the cell proliferation rate, the effect of the chitosan-sodium carbonate complex was assessed in this cell line by using the MTT assay [28,29].

**Figure 6:**
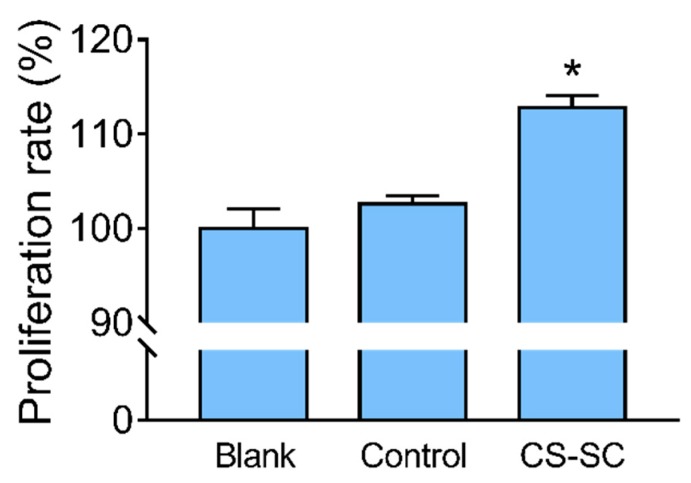
Percentage of cell proliferation after 48 h exposure. * Statistically significant differences were observed between the control film and CS-SC film wells (values are means ± s.e.m, *p* < 0.05, *n* = 3).

Due to the porous film structure and biocompatibility, we believed that the film could promote cell proliferation [30]. The growth of a fibroblast cell culture was evaluated using the MTT assay [31] after 48 h exposure. Figure 6 shows the percentage of cell proliferation, determined as follows: (OD of experimental well/OD of blank well) × 100%. 

Cell viability was not significantly changed after exposure of L929 to the control film. As demonstrated in Figure 6, a statistically significant difference (*p* < 0.05) between the percentages of cell proliferation in the wells treated with CS-SC film relative to the control film was observed, indicating that the CS-SC film promoted cell proliferation. 

**Figure 7:**
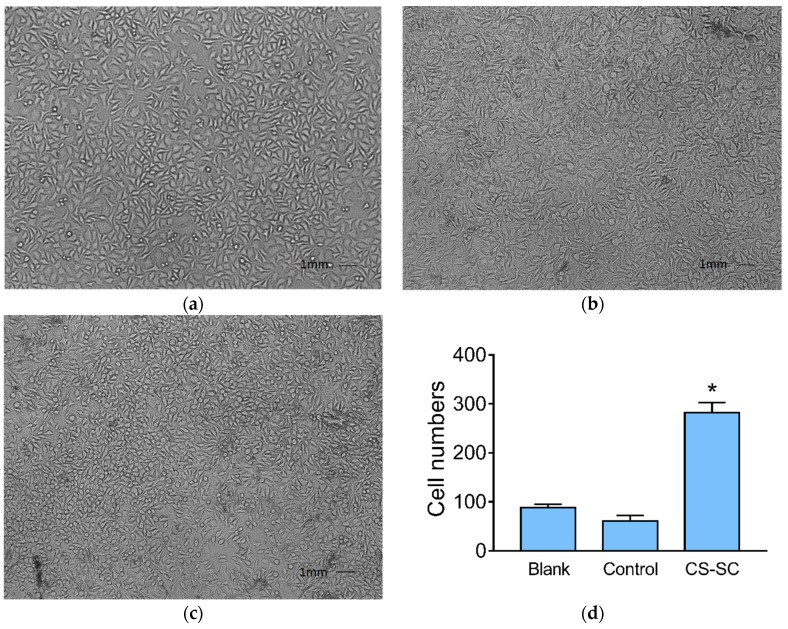
Morphology changes in L929 fibroblast cells in different wells and the statistical result of round cells. (**a**) Blank; (**b**) control; (**c**) CS-SC; (**d**) statistical result of round cells (values are means ± s.e.m, *p* < 0.05, *n* = 3).

The morphology of L929 fibroblast cells was observed by an inverted microscope. As shown in Figure 7a, the cells in the blank wells were tightly connected and had an irregular triangular or spindle shape. There were some non-adhesive cells in the wells containing control films suspended in the culture medium. The number of cells growing in the wells containing the complex films increased significantly, but the morphology of the cells changed slightly; the number of round cells was greater than that in the blank wells (*p* < 0.05) (Figure 7d).

## 3. Materials and Methods

### 3.1. Materials and Cell Line

Chitosan was purchased from Sinopharm Chemical Reagent Co., Ltd. (Shanghai, China). The degree of deacetylation was 90% and viscosity-average molecular weight was 5.3 × 10^4^. Other reagents, such as sodium carbonate, acetic acid, ethanol, potassium bromide (KBr), and solvents, were analytical grade and were also supplied by Sinopharm Chemical Reagent Co., Ltd. (Shanghai, China). FBS (fetal bovine serum) and Dulbecco’s Modified Eagle Medium (DMEM) culture medium were purchased from Gibco Company (Langley, OK, USA). Penicillin–streptomycin for the cell culture, as well as trypsin, were purchased from Beyotime Bio Technology Co., Ltd. (Shanghai, China). MTT (M2128) was supplied by Sigma Company (St. Louis, MO, USA).

Fibroblasts, NCTC clone 929 (mouse connective tissue), were purchased from Cell Resources Center (Shanghai Academy of Life Sciences, Chinese Academy of Sciences, Shanghai, China) and preserved in the School of Pharmaceutical Sciences, Jiangnan University. L929 cells were grown in DMEM supplemented with 10% (*v*/*v*) FBS and 100 IU/mL penicillin–streptomycin. The cells were maintained in a humid incubator (5% CO_2_) at 37 °C [32].

### 3.2. Instruments

The structure of the complex was determined by FT-IR spectroscopy (Thermo Nicolet Corporation, NEXUS, Madison, WI, USA), SSNMR spectrometry with a superconducting magnet (Brook Corporation, Advance III/WB-400, Tübingen, Germany) and EDS (Ametek Corporation, TEAM Octane Super, San Diego, CA, USA) equipped with SEM (Hitachi Corporation, S4800, Tokyo, Japan). The crystalline structure of the complex and morphology of the microporous films were studied by SEM (FEI Corporation, Quanta 200, Hillsboro, OR, USA). Cytotoxicity experiments were carried out using a microplate reader (MD Corporation, Spectra MAX M2e, San Jose, CA, USA) and an inverted microscope (Olympus Corporation, CKX 41, Tokyo, Japan).

### 3.3. Experimental Procedure

#### 3.3.1. Preparation of the Chitosan-Sodium Carbonate Complex

One gram of chitosan was added to 100 mL of water in a beaker and stirred for 1 min. One milliliter of glacial acetic acid (CAS:64-19-7) was added and stirred until completely dissolved. Sodium carbonate solution (50 mL of 0.5 mol/L) was rapidly added, which produced a large number of bubbles, and the solution was stirred until most of the bubbles vanished. The solution was viscous and was used to prepare the films (Section 3.3.2).

The chitosan-sodium carbonate complex was obtained by adding absolute ethanol to the beaker, which produced a white floc. The volume of absolute ethanol was 3× (*v*/*v*) that of the aforementioned solution. Then, the floc was vacuum-filtered and freeze-dried.

The chitosan-sodium carbonate complex can be returned to chitosan by treatments such as dialysis, using acid to adjust the pH to neutral, and washing with a certain concentration of ethanol. In this study, we prepared the restored chitosan by adjusting the complex solution pH to neutral with hydrochloric acid, and then freeze-dried the gelatinous precipitate.

#### 3.3.2. Preparation of the Polymer Films

To each well of a 96-well plate, 100 μL of the complex solution was added, spread evenly, and washed with 50% ethanol (*v*/*v*), thus forming a layer of film. The solvent was evaporated in the oven at 80 °C for 1 h. The film was then soaked in 75% ethanol (*v*/*v*) for 12 h and washed with phosphate buffered saline (PBS) three times.

The control film was prepared as follows: 1 g of chitosan was dissolved in 1% acetic acid solution (*v*/*v*). A total of 100 μL of the chitosan/acetic acid solution was added to the well, spread evenly, and soaked in a 0.1 mol/L sodium hydroxide solution until a layer of film was formed [14]. The solvent was evaporated in the oven at 80 °C for 1 h. The film was then soaked in 75% ethanol (*v*/*v*) for 12 h and washed with PBS three times.

#### 3.3.3. Schiff Base Reaction

A total of 1 g of the chitosan-sodium carbonate complex was dissolved in 50 mL of deionized water, and 1.0 mL of glutaraldehyde (50% *v*/*v*) was dissolved in 20 mL of deionized water. The two solutions were mixed under vigorous stirring for 10 min [25].

#### 3.3.4. FT-IR

The KBr method was used [20]. The scanning range was 400–4000 cm^−1^, with step length 1 cm^−1^, and scanning was performed 32 times. 

#### 3.3.5. NMR

SSNMR experiments were performed on a Bruker Advance III HD 400 spectrometer (Tübingen, Germany) operating at a Larmor frequency of 400.25 MHz for 1H and 100.64 MHz for ^13^C; the spectrometer was equipped with an H/F/X triple-resonance magic-angle spinning (MAS) probe, supporting MAS rotors with an outer diameter of 3.2 mm. The rf-nutation frequency for ^1^H and ^13^C was 78.1 kHz, corresponding to 3.2 μs and a 90° pulse. 1D ^13^C{^1^H} cross-polarization/magic-angle spinning (CP/MAS) spectra were recorded using a CP contact time of 2 ms, a recycle delay of 5 s, and 1024 scans, with SPINAL-64 decoupling applied during acquisition [33]. The 2D ^13^C–^1^H FSLG-HECTOR [34] experiments used a MAS frequency of 10.0 kHz, a recycle delay of 3 s, and a cross-polarization (CP) time of 0.5 ms at 100 scans for a total of 70 t1 increments. Each t1 increment had a span of one basic FSLG block (30.21 μs), [35], and high-power ^1^H SPINAL-64 decoupling was used during acquisition. A scaling factor of 0.570 was determined by recording a 2D spectrum for adamantane using identical experimental conditions to rescale the indirect dimension for the investigated samples. The chemical shifts are referenced with respect to tetramethyl silane (TMS), using adamantane as the second standard (^1^H, δ = 1.85 ppm; ^13^C, δ = 38.49 ppm).

#### 3.3.6. SEM

The prepared film was freeze-dried, cut into strips, or torn off from the surface to observe the internal structure. After surface sputtering, the morphology and structure were observed by SEM [36].

#### 3.3.7. Cytotoxicity Study

The MTT method was used to analyze the effect of the film formed by the novel complex on the relative cell proliferation ratio of L929 rat fibroblasts and cytotoxicity. The preparation of the films was the same as that detailed in Section 3.3.2. Briefly, L929 cells were seeded into 96-well plates (1 × 10^5^ cells per well) and incubated for 48 h. To each well, 20 μL of 5 mg/mL MTT in PBS was added and then cultured for 4 h. Finally, 100 μL of DMSO was added instead of the above medium to dissolve the formazan crystals. OD was determined at 570 nm by using a microplate reader [37]. Cell growth and viability were observed and photographed using an inverted microscope.

#### 3.3.8. Statistical Analysis

The results are expressed as means ± s.e.m. Statistical analysis was performed using one-way ANOVA followed by Newman–Keuls post hoc tests. All the analyses were conducted using GraphPad Prism 5 (GraphPad Software, Inc., La jolla, CA, USA), and, if *p* < 0.05, results were considered statistically significant.

## 4. Conclusions

A water-soluble complex of chitosan–sodium carbonate is reported, which was obtained by adding a large amount of sodium carbonate to chitosan/acetic acid solution. FT-IR, EDS, and SEM findings support the formation of carbonate crystallization in the complex. Furthermore, SSNMR ^13^C{^1^H} CP confirmed the association between the ammonium cation and carbonate anions. The complex could be returned to chitosan by soaking in an alcohol solution, and the film maintained good biocompatibility. We showed, for the first time, that this new CS-SC film may promote cell proliferation, making it a candidate material for tissue engineering. Furthermore, greater focus should be directed toward the principle of dissolution and the solubility of the chitosan–sodium carbonate complex at different degrees of deacetylation and from different sources.

## Figures and Tables

**Table 1:** Atomic percentage of elements C, N, O, and Na.

Element	Atomic Percentage		
	A	B	C
C	61.99	33.87	53.62
N	7.92	2.14	9.66
O	30.09	48.91	34.24
Na	0.00	15.08	2.49

## References

[1] Wu Q.-X., Lin D.-Q., Yao S.-J. (2014). Design of Chitosan and Its Water Soluble Derivatives-Based Drug Carriers with Polyelectrolyte Complexes. Mar. Drugs.

[2] Yang S., Shao D., Wang X., Hou G., Nagatsu M., Tan X., Ren X., Yu J. (2015). Design of Chitosan-Grafted Carbon Nanotubes: Evaluation of How the –OH Functional Group Affects Cs^+^ Adsorption. Mar. Drugs.

[3] Younes I., Rinaudo M. (2015). Chitin and Chitosan Preparation from Marine Sources. Structure, Properties and Applications. Mar. Drugs.

[4] Jiang Y., Fu C., Wu S., Liu G., Guo J., Su Z. (2017). Determination of the Deacetylation Degree of Chitooligosaccharides. Mar. Drugs.

[5] Mourya V.K., Inamdar N.N. (2008). Chitosan-modifications and applications: Opportunities galore. React. Funct. Polym..

[6] Riva R., Ragelle H., des Rieux A., Duhem N., Jérôme C., Véronique Préat V., Jayakumar R., Prabaharan M., Muzzarelli R.A.A. (2011). Chitosan and chitosan derivatives in drug delivery and tissue engineering. Chitosan for Biomaterials II..

[7] de Queiroz Antonino R.S.C.M., Lia Fook B.R.P., de Oliveira Lima V.A., de Farias Rached R.Í., Lima E.P.N., da Silva Lima R.J., Peniche Covas C.A., Lia Fook M.V. (2017). Preparation and Characterization of Chitosan Obtained from Shells of Shrimp (*Litopenaeus vannamei* Boone). Mar. Drugs.

[8] Kulkarni A.D., Patel H.M., Surana S.J., Vanjari Y.H., Belgamwar V.S., Pardeshi C.V. (2017). *N,N,N*-Trimethyl chitosan: An advanced polymer with myriad of opportunities in nanomedicine. Carbohydr. Polym..

[9] Sun Z., Shi C., Wang X., Fang Q., Huang J. (2017). Synthesis, characterization, and antimicrobial activities of sulfonated chitosan. Carbohydr. Polym..

[10] Kamari A., Aljafree N.F.A., Yusoff S.N.M. (2016). *N,N*-dimethylhexadecyl carboxymethyl chitosan as a potential carrier agent for rotenone. Int. J. Biol. Macromol..

[11] Straccia M.C., d’Ayala G.G., Romano I., Oliva A., Laurienzo P. (2015). Alginate Hydrogels Coated with Chitosan for Wound Dressing. Mar. Drugs.

[12] Bellich B., D’Agostino I., Semeraro S., Gamini A., Cesàro A. (2016). “The Good, the Bad and the Ugly” of Chitosans. Mar. Drugs.

[13] Varum K.M., Ottoy M.H., Smidsrod O. (2001). Acid hydrolysis of chitosans. Carbohydr. Polym..

[14] Duan J., Liang X., Cao Y., Wang S., Zhang L. (2015). High strength chitosan hydrogels with biocompatibility via new avenue based on constructing nanofibrous architecture. Macromolecules.

[15] Fang Y., Zhang R., Duan B., Liu M., Lu A., Zhang L. (2017). Recyclable universal solvents for chitin to chitosan with various degrees of acetylation and construction of robust hydrogels. ACS Sustain. Chem. Eng..

[16] Fang Y., Duan B., Lu A., Liu M., Liu H., Xu X., Zhang L. (2015). Intermolecular interaction and the extended wormlike chain conformation of chitin in naoh/urea aqueous solution. Biomacromolecules.

[17] Nishimura T., Ito T., Yamamoto Y., Yoshio M., Kato T. (2008). Macroscopically ordered polymer/CaCO3 hybrids prepared by using a liquid-crystalline template. Angew. Chem. Int. Ed..

[18] Piron E., Domard A. (1998). Formation of a ternary complex between chitosan and ion pairs of strontium carbonate. Int. J. Biol. Macromol..

[19] Fu R., Ji X., Ren Y., Wang G., Cheng B. (2017). Antibacterial blend films of cellulose and chitosan prepared from binary ionic liquid system. Fibers Polym..

[20] Silva D.S., Almeida A., Prezotti F., Cury B., Campana-Filho S.P., Sarmento B. (2017). Synthesis and characterization of 3,6-O,O′-dimyristoyl chitosan micelles for oral delivery of paclitaxel. Colloids Surf. B.

[21] Taleb M.F., Alkahtani A., Mohamed S.K. (2015). Radiation synthesis and characterization of sodium alginate/chitosan/hydroxyapatite nanocomposite hydrogels: A drug delivery system for liver cancer. K. Mohamed, Polym. Bull..

[22] Roomans G.M., Dragomir A. (2014). X-ray microanalysis in the scanning electron microscope. J. Electron Microsc..

[23] Xing Q. (2016). Information or resolution: Which is required from an SEM to study bulk inorganic materials?. Scanning.

[24] Peng S., Meng H.-C., Zhou L., Chang J. (2014). Synthesis of Novel Magnetic Cellulose-Chitosan Composite Microspheres and Their Application in Laccase Immobilization. J. Nanosci. Nanotechnol..

[25] Chang X.H., Chen D.R., Jiao X.L. (2008). Chitosan-based aerogels with high adsorption performance. J. Phys. Chem. B.

[26] King C., Stein R.S., Shamshina J.L., Rogers R.D. (2017). Measuring the Purity of Chitin with a Clean, Quantitative Solid-State NMR Method. ACS Sustain. Chem. Eng..

[27] Oliveira J.R., Martins M.C.L., Mafra L., Gomes P. (2012). Synthesis of an O-alkynyl-chitosan and its chemoselective conjugation with a PEG-like amino-azide through click chemistry. Carbohydr. Polym..

[28] Zhao Y., Wang Z., Zhang Q., Chen F., Yue Z., Zhang T., Deng H., Huselstein C., Anderson D.P., Chang P.R. (2018). Accelerated skin wound healing by soy protein isolate-modified hydroxypropyl chitosan composite films. Int. J. Biol. Macromol..

[29] Shanmugapriya K., Kim H., Saravana P.S., Chun B.-S., Kang H.W. (2018). Fabrication of multifunctional chitosan-based nanocomposite film with rapid healing and antibacterial effect for wound management. Int. J. Biol. Macromol..

[30] Gómez Chabala L.F., Cuartas C.E.E., López M.E.L. (2017). Release Behavior and Antibacterial Activity of Chitosan/Alginate Blends with *Aloe vera* and Silver Nanoparticles. Mar. Drugs.

[31] Zhang Q.C., Luo H., Zhang Y., Zhou Y., Ye Z., Tan W., Lang M. (2013). Fabrication of three-dimensional poly(epsilon-caprolactone) scaffolds with hierarchical pore structures for tissue engineering. Mater. Sci. Eng. C.

[32] Kanimozhi K., Basha S.K., Kumari V.S., Kaviyarasu K., Maaza M. (2018). In vitro cytocompatibility of chitosan/PVA/methylcellulose - Nanocellulose nanocomposites scaffolds using L929 fibroblast cells. Appl. Surf. Sci..

[33] Fung B.M., Khitrin A.K., Ermolaev K. (2000). An improved broadband decoupling sequence for liquid crystals and solids. J. Magn. Reson..

[34] Van Rossum B.J., Forster H., de Groot H.J.M. (1997). High-field and high-speed CP-MAS C-13 NMR heteronuclear dipolar-correlation spectroscopy of solids with frequency-switched Lee-Goldburg homonuclear decoupling. J. Magn. Reson..

[35] Ladizhansky V., Vega S. (2000). Polarization transfer dynamics in Lee-Goldburg cross polarization nuclear magnetic resonance experiments on rotating solids. J. Chem. Phys..

[36] Szymańska E., Szekalska M., Czarnomysy R., Lavrič Z., Srčič S., Miltyk W., Winnicka K. (2016). Novel Spray Dried Glycerol 2-Phosphate Cross-Linked Chitosan Microparticulate Vaginal Delivery System—Development, Characterization and Cytotoxicity Studies. Mar. Drugs.

[37] Magesh G., Bhoopathi G., Nithya N., Arun A.P., Ranjith Kumar E. (2018). Structural, morphological, optical and biological properties of pure ZnO and agar/zinc oxide nanocomposites. Int. J. Biol. Macromol..

